# Cost-utility analysis of add-on ezetimibe to moderate-intensity statin versus moderate-intensity statin alone for secondary prevention in patients with acute coronary syndrome intolerant to high-intensity statin therapy in Thailand

**DOI:** 10.3389/fphar.2026.1813614

**Published:** 2026-05-20

**Authors:** Nattiwat Promchit, Surarong Chinwong, Unchalee Permsuwan

**Affiliations:** 1 Master’s Degree Program in Clinical Pharmacy, Faculty of Pharmacy, Chiang Mai University, Chiang Mai, Thailand; 2 Department of Pharmaceutical Care, Faculty of Pharmacy, Chiang Mai University, Chiang Mai, Thailand; 3 Research Center for Innovation in Analytical Science and Technology for Biodiversity-Based Economic and Society (I-ANALY-S-T_B.BES-CMU), Multidisciplinary Research Institute (MDRI), Chiang Mai University, Chiang Mai, Thailand; 4 Center for Medical and Health Technology Assessment (CM-HTA), Department of Pharmaceutical Care, Faculty of Pharmacy, Chiang Mai University, Chiang Mai, Thailand

**Keywords:** acute coronary syndrome, cost-utility analysis, ezetimibe, statin, Thailand

## Abstract

**Background:**

Although high-intensity statin therapy is recommended for the secondary prevention of acute coronary syndrome (ACS), a subset of patients is unable to tolerate such regimens due to statin-associated adverse effects. For these patients, adding ezetimibe to a maximally tolerated moderate-intensity statin is a guideline-recommended lipid-lowering strategy. This study aimed to evaluate the cost-utility of ezetimibe added to moderate-intensity statin therapy, compared with moderate-intensity statin therapy alone, for the secondary prevention of ACS in Thailand.

**Methods:**

A Markov model with four health states was developed. Incremental cost-effectiveness ratios (ICERs) were estimated to compare strategies from both societal and healthcare provider perspectives. Transition probabilities, utility values, and cost inputs were derived from the IMPROVE-IT trial and relevant literature, supplemented with Thailand-specific data. Costs (2024 Thai baht [THB]) and outcomes were discounted at an annual rate of 3%. One-way and probabilistic sensitivity analyses were conducted. The intervention was considered cost-effective if the ICER was below the Thai willingness-to-pay (WTP) threshold of 160,000 THB per QALY (4,533 USD/QALY).

**Results:**

Ezetimibe added to moderate-intensity statin therapy yielded an ICER of 155,312 THB/QALY (4,400.4 USD/QALY) from the societal perspective and 148,934 THB/QALY (4,219.7 USD/QALY) from the healthcare provider perspective; both were below the 160,000 THB/QALY threshold. In the one-way sensitivity analysis, the relative risk of death from myocardial infarction (MI) associated with ezetimibe was the most influential parameter (ICER change: −27.31% to +54.32%). Probabilistic sensitivity analysis indicated a 56.8% probability of cost-effectiveness at the predefined threshold.

**Conclusion:**

These findings suggest that adding ezetimibe to moderate-intensity statin therapy may be cost-effective compared with moderate-intensity statin monotherapy for the secondary prevention of ACS among patients in Thailand who are intolerant to high-intensity statins. However, because the base-case ICER was only marginally below the national threshold and probabilistic sensitivity analysis showed a 56.8% probability of cost-effectiveness, the results should be interpreted with appropriate caution.

## Background

Acute coronary syndrome (ACS) is a common cardiovascular condition associated with high mortality in Thailand, with an increasing burden over time (The Heart Association of Thailand, 2020). Recent national data indicate that the hospitalization rate for ACS was 31.76 per 100,000 population, and the 30-day mortality rates per 100 patients after admission from 2020 to 2022 were 16.41, 16.95, and 17.07, respectively (Resource information: ST-segment elevation myocardial infarction STEMI, 2023). Among lipid abnormalities associated with cardiovascular disease, elevated low-density lipoprotein cholesterol (LDL-C) is a major modifiable risk factor and remains a primary therapeutic target (Collet et al., 2021).

The 2019 ESC/EAS guidelines recommend high-intensity statin therapy for the secondary prevention of ACS in patients without contraindications or documented intolerance (Mach et al., 2020). The Thai Acute Coronary Syndromes Guidelines similarly recommend high-intensity statins to lower LDL-C in patients with ACS (The Heart Association of Thailand, 2020). However, real-world data suggest that high-intensity statin therapy is underutilized in Thai practice. The Dyslipidemia International Study (DYSIS) II reported that Thai patients with ACS received statin doses equivalent to a mean of 17 ± 13 mg/day of atorvastatin (Buddhari et al., 2020), which is substantially below high-intensity dosing (e.g., atorvastatin 40 mg or rosuvastatin 20 mg) (Collet et al., 2021). Consistent with this treatment pattern, LDL-C control is often suboptimal; mean LDL-C levels of 106.2 ± 39.4 mg/dL exceed the recommended target of <70 mg/dL (The Heart Association of Thailand, 2020). Intolerance and adverse effects related to high-intensity statin therapy contribute to this gap, limiting its use in a subset of patients and resulting in the routine use of moderate-intensity statins in clinical practice (Buddhari et al., 2020). In one report, the primary reason for discontinuing statin therapy was adverse effects, with muscle-related symptoms reported in 29% of patients (Cohen et al., 2012). Studies in Thailand further suggest that approximately 10% of individuals receiving high-intensity statin therapy discontinue therapy or demonstrate poor adherence due to statin-associated muscle symptoms (SAMS) (e.g., myalgia, myopathy, and rhabdomyolysis) and potential drug–drug interactions (e.g., with fibrates, gemfibrozil, and colchicine) (Supsongserm et al., 2013; Boonmuang et al., 2013).

For patients who are intolerant to high-intensity statin therapy, the 2019 ESC/EAS guidelines recommend adding a non-statin lipid-modifying agent to the maximally tolerated statin dose (Mach et al., 2020). Evidence also supports LDL-C target attainment as a pragmatic approach in this population. In the LODESTAR trial, a treat-to-target strategy (LDL-C 50–70 mg/dL) in patients with coronary artery disease was non-inferior to high-intensity statin therapy over 3 years in preventing a composite endpoint of death, myocardial infarction, stroke, or coronary revascularization (Hong et al., 2023). These findings underscore the clinical importance of achieving LDL-C targets rather than focusing solely on statin intensity.

Ezetimibe inhibits intestinal cholesterol absorption and is recommended as an adjunctive (non-statin) therapy to further reduce LDL-C in the secondary prevention of ACS (The Heart Association of Thailand, 2020; Collet et al., 2021). The Improved Reduction of Outcomes: Vytorin Efficacy International Trial (IMPROVE-IT) compared simvastatin 40 mg plus ezetimibe 10 mg with simvastatin 40 mg alone and demonstrated greater LDL-C reduction and improved cardiovascular outcomes with combination therapy in patients after ACS. At 7 years, the Kaplan–Meier event rate for the primary endpoint was 32.7% in the combination group versus 34.7% in the monotherapy group (Cannon et al., 2015). Other studies have similarly shown that adding ezetimibe to statin therapy enhances LDL-C reduction and may reduce the risk of subsequent events, including myocardial infarction (MI), stroke, and death (Reckless et al., 2008; Brudi et al., 2009; Hagiwara et al., 2017; Tan et al., 2021). Therefore, for patients who cannot achieve guideline-recommended LDL-C targets due to high-intensity statin intolerance, ezetimibe added to a maximally tolerated statin represents a clinically feasible, evidence-based lipid-lowering strategy (The Heart Association of Thailand, 2020; Mach et al., 2020).

Until 2024, the Thai National List of Essential Medicines (NLEM) included four statin formulations: simvastatin (10, 20, and 40 mg) and atorvastatin (40 mg). Within this list, atorvastatin 40 mg was the only high-intensity statin option (National list of essential medicines NLEM, 2024). In 2024, ezetimibe was added to the NLEM with an indication as first-line therapy for sitosterolemia; however, additional economic evidence is required to support expansion to other indications (National list of essential medicines NLEM, 2024). Prior economic evaluations from the United States, the United Kingdom, and China have suggested benefits of combining ezetimibe with statin therapy for secondary prevention in patients with ACS (Cannon et al., 2015; Reckless et al., 2008; Ran et al., 2017). Therefore, this study aimed to evaluate the cost-utility of ezetimibe added to moderate-intensity statin therapy versus moderate-intensity statin therapy alone for secondary prevention among patients with ACS who are intolerant to high-intensity statin therapy in Thailand.

## Methods

To address the primary objective of this study, we conducted a model-based cost-utility analysis. Sensitivity analyses were performed to assess the robustness of the results. The methods were consistent with the Thai Health Technology Assessment (HTA) Guideline (Author anonymous, 2013).

### Model structure

**FIGURE 1:**
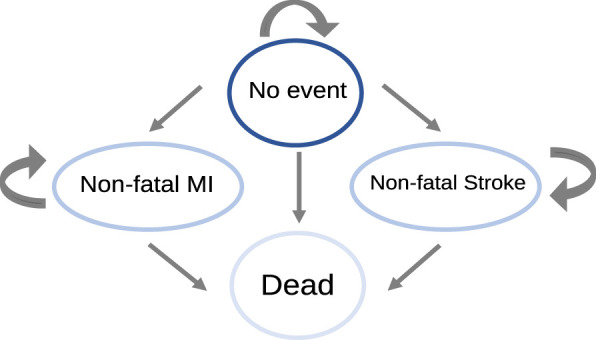
Markov model. MI, myocardial infarction. Figure adapted from Nikolic et al. (2013).

We adapted a previously published Markov model for ACS (Nikolic et al., 2013). In brief, the model comprised four mutually exclusive health states: (1) no further event, (2) non-fatal myocardial infarction (MI), (3) non-fatal stroke, and (4) death (Figure 1). Patients entered the model in the “no further event” state. During each annual cycle, patients could remain in the same state or transition to non-fatal MI, non-fatal stroke, or death. After entering the non-fatal MI or non-fatal stroke state, patients could remain in that state in subsequent cycles until transitioning to death. The cycle length was 1 year, and the model used a lifetime horizon. The base-case cohort had a mean starting age of 62 years, based on the average age of patients with ACS reported in the Thai Registry (THAI ACS REGISTRY, 2023).

Several simplifying assumptions were applied. First, recurrent cardiovascular events were not modeled beyond the first post-event state, consistent with the source model (Nikolic et al., 2013), and crossover transitions between the non-fatal MI and non-fatal stroke states were not included. Direct Thai data on the rate of such sequential transitions among statin-treated ACS patients are unavailable. However, the IMPROVE-IT trial reported a 7-year cumulative incidence of ischemic stroke of approximately 4.1% among statin-treated ACS patients, corresponding to an average annual risk of 0.59% (Bohula et al., 2017); this value can be considered a plausible upper bound for the omitted crossover transition. Because the same assumption was applied to both treatment arms, the impact on incremental outcomes is expected to be minimal. Second, concurrent MI and stroke within the same cycle were not modeled, given the low reported incidence of co-occurrence (approximately 1.6%) (Aggarwal et al., 2021; Alqahtani et al., 2017). Third, treatment discontinuation and adverse events were not explicitly modeled because the tolerability profile of ezetimibe is comparable to that of statin monotherapy (Cannon et al., 2015). Overall, these assumptions are expected to have minimal effects on incremental outcomes. However, the direction of bias may vary across assumptions. Omitting recurrent cardiovascular events may underestimate both future event-related costs and the long-term treatment benefits of ezetimibe; therefore, the net direction of bias on the incremental comparison remains uncertain. In contrast, the remaining assumptions, which exclude concurrent MI and stroke, treatment discontinuation, and adverse events, are expected to have non-differential effects across both arms, with minimal impact on the incremental cost-effectiveness ratio.

### Intervention and comparator

The intervention was ezetimibe 10 mg once daily added to moderate-intensity statin therapy. The comparator was moderate-intensity statin therapy alone. Simvastatin 40 mg once daily was selected to represent a maximally tolerated statin regimen among patients intolerant to high-intensity statin therapy. This choice was supported by its inclusion on the Thai National List of Essential Medicines (NLEM) (National list of essential medicines NLEM, 2024) and its use as the comparator in the IMPROVE-IT trial (Cannon et al., 2015).

### Input parameters

All model inputs are summarized in Table 1. Parameters were categorized by derivation: directly observed values (a) were obtained from published sources without mathematical transformation, whereas derived values (b) were calculated (e.g., conversion of rates to probabilities) or updated to 2024 values using inflation indices.

**TABLE 1:** Key input parameters.

Parameter description	Base-case value	Interval	Distribution	Sources
Moderate-intensity statin alone				
Probability of developing MI^a^	0.016900	0.0152–0.0186	Beta	Cannon (Cannon et al., 2015)
Probability of developing stroke^a^	0.005415	0.0049–0.006	Beta	Cannon (Cannon et al., 2015)
Probability of death from MI^b^	0.090263	0.0812–0.0993	Beta	Thai ACS Registry (UPDATE Thai ACS Registry for Save Thais, 2024)
Probability of death from stroke^b^	0.071593	0.0644–0.0788	Beta	DMS (Health Information System, 2024)
Ezetimibe + Moderate-intensity statin				
Probability of developing MI^a^	0.014779	0.0133–0.0163	Beta	Cannon (Cannon et al., 2015)
Probability of developing stroke^a^	0.004653	0.0042–0.0051	Beta	Cannon (Cannon et al., 2015)
Probability of death from MI^b^	0.090263	0.0812–0.0993	Beta	Thai ACS Registry (UPDATE Thai ACS Registry for Save Thais, 2024)
Probability of death from stroke^b^	0.071593	0.0644–0.0788	Beta	DMS (Health Information System, 2024)
Markov model				
Log hazard ratio for excess mortality in no-event state^b^ ^,c^	0.693	0.62–0.76	Log-normal	Nikolic (Nikolic et al., 2013)
Relative risk of death in MI^a^	0.840	0.55–1.07	Log-normal	Cannon (Cannon et al., 2015)
Relative risk of death in stroke^a^	0.900	0.84–0.96	Log-normal	Cannon (Cannon et al., 2015)
Relative risk of death from any cause^a^	0.990	0.91–1.07	Log-normal	Cannon (Cannon et al., 2015)
Direct medical cost (THB (USD))				
Drug cost (THB (USD))				
Cost of moderate-intensity statin^b^	292 (8.3)	234–350 (6.6–9.9)	Gamma	DMSIC (National Drug Prices, 2023)
Cost of ezetimibe^b^	1,730 (49.0)	1,384–2,075 (39.2–58.8)	Gamma	DMSIC (National Drug Prices, 2023)
Treatment cost (THB (USD))				
ACS first year^b^	65,507 (1,856)	45,174–67,762 (1,279.9–1,919.9)	Gamma	Anukoolsawat (Anukoolsawat et al., 2006)
ACS second year and onward^b^	39,617 (1122.4)	27,320–40,980 (774–1,161.1)	Gamma	Anukoolsawat (Anukoolsawat et al., 2006)
Non-fatal MI first-year^b^	148,366 (4,203.6)	102,314–153,472 (2,898.8–4,348.2)	Gamma	Tamteeranon (Tamteeranon et al., 2008)
Non-fatal MI second year and onward^b^	15,924 (451.2)	10,982–16,472 (311.1–466.7)	Gamma	Tamteeranon (Tamteeranon et al., 2008)
Non-fatal stroke first year^b^	73,440 (2,080.7)	50,645–75,967 (1,434.9–2,152.3)	Gamma	Tamteeranon (Tamteeranon et al., 2008)
Non-fatal stroke in the second year and onward^b^	11,634 (329.6)	8,023–12,035 (227.3–341)	Gamma	Tamteeranon (Tamteeranon et al., 2008)
Direct non-medical cost (THB (USD))				
Direct non-medical cost first year^b^	3,730 (105.7)	2,572–3,858 (72.9–109.3)	Gamma	Anukoolsawat (Anukoolsawat et al., 2006)
Direct non-medical costs from the second year onward^b^	5,394 (152.8)	3,720–5,580 (105.4–158.1)	Gamma	Anukoolsawat (Anukoolsawat et al., 2006)
Utility				
Utility of ACS^a^	0.82	0.738–0.902	Beta	Gencer (Gencer et al., 2016)
Annual utility decrement after MI^a^	0.41635	0.3747–0.458	Gamma	Tamteeranon (Tamteeranon et al., 2008)
Annual utility decrement after stroke^a^	0.2259	0.2033–0.2485	Gamma	Tamteeranon (Tamteeranon et al., 2008)

^a^ Directly observed values obtained from published trial or survey data without mathematical transformation.

^b^ Derived values transformed from source data using the formula p = 1 - exp (-rt) or adjusted using the consumer price index.

^C^ Derived as ln(HR) = ln(2) = 0.693, where HR = 2.0 represents the hazard ratio for ACS-related excess mortality reported by Nikolic et al. (2013).

Costs are presented in 2024 Thai baht (THB), with USD, values in parentheses, calculated at an exchange rate of 35.2952 THB/USD.

ACS, acute coronary syndrome; DMS, department of medical sciences; DMSIC, drug and medical supply information center; MI, myocardial infarction; THB, thai baht.

### Transition probabilities

Transition probabilities for non-fatal MI and non-fatal stroke were derived from event rates reported in the IMPROVE-IT trial (Cannon et al., 2015). For mortality, age-specific mortality rates (ASMR) from the Thai life tables (Life tables: Life tables by country Thailand, 2020) were converted to annual transition probabilities using the formula *p = 1 - exp(-rt)*, where *p* is the annual probability, *r* is the mortality rate, and *t* is the cycle length (1 year). Background mortality probabilities were then adjusted using the hazard ratio for ACS-related mortality reported by Nikolic et al. (THAI ACS REGISTRY, 2023) to reflect excess mortality risk after ACS. Mortality following non-fatal MI and non-fatal stroke was obtained from the Thai ACS Registry (UPDATE Thai ACS Registry for Save Thais, 2024, 2024) and the Health Information System (Health Information System, 2024), respectively. Relative risks associated with ezetimibe add-on therapy from IMPROVE-IT (Cannon et al., 2015) were applied to relevant transition probabilities to estimate outcomes in the ezetimibe arm.

### Costs

The analysis was conducted from both societal and healthcare provider perspectives. Direct medical costs included costs related to ACS, MI, and stroke. Direct non-medical costs included transportation and caregiver time. Direct non-medical costs were included in the societal perspective but excluded from the healthcare provider perspective. In accordance with Thai HTA guidelines, indirect costs were not included in this cost-utility analysis (Author anonymous, 2013).

Drug costs for simvastatin 40 mg (moderate-intensity statin) and ezetimibe were obtained from the Drug and Medical Supply Information Center (DMSIC) (National Drug Prices, 2023). In line with Thai HTA guidelines, the median cost across available brands was used (Author anonymous, 2013). The median daily cost of simvastatin was 0.80 THB/day (0.02 USD), and the median daily cost of ezetimibe was 4.74 THB/day (0.13 USD) (Supplementary Appendix 2). Costs for the first year after ACS, subsequent years after ACS, and direct non-medical costs were obtained from the Thai ACS study by Anukoolsawat et al. (2006). Costs for non-fatal MI and non-fatal stroke were obtained from the survey by Tamteeranon et al. (2008). All cost inputs were inflated to 2024 values using the medical-care consumer price index (Consumer Price Index CPI, 2026). Costs are reported in Thai baht (THB); the 2024 average exchange rate was 1 USD = 35.2952 THB (Author anoymous, 2025).

### Utility

Quality-adjusted life-years (QALYs) were calculated as the product of utility and life-years. Baseline utility for the “no further event” state after ACS was obtained from EQ-5D data reported by Gencer et al. (2016), who assessed patients 1 year after ACS using the EuroQol five-dimension questionnaire (EQ-5D) and the visual analog scale (VAS). Utility decrements associated with MI and stroke were based on a Thailand-specific study by Tamteeranon et al. (2008).

### Model analysis

Lifetime costs and QALYs were estimated for each strategy and summarized as the incremental cost-effectiveness ratio (ICER), defined as incremental costs divided by incremental QALYs. In the base-case analysis, costs and outcomes were discounted at 3% annually, consistent with Thai HTA recommendations (Author anonymous, 2013). The intervention was considered cost-effective if the ICER was below the Thai willingness-to-pay threshold (ceiling ratio) of 160,000 THB/QALY (4,533 USD/QALY) gained (Thavorncharoensap et al., 2013). The model was implemented in Microsoft Excel (Microsoft Corporation, Redmond, WA, USA). A half-cycle correction was applied to account for the fact that transitions between states are expected to occur on average in the middle of each cycle (Briggs et al., 2006).

### Sensitivity analyses

We conducted sensitivity analyses to evaluate parameter uncertainty. One-way sensitivity analysis assessed the impact of varying each parameter individually (probabilities, relative risks [RRs], costs, and utilities) while holding other inputs constant. When available, parameter ranges were based on 95% confidence intervals (CIs) or standard errors. When such data were unavailable, transition probabilities were varied by ±10% and costs by ±20%. Results were summarized in a tornado diagram.

Probabilistic sensitivity analysis (PSA) was conducted to jointly propagate uncertainty across all parameters using Monte Carlo simulation. Each parameter was assigned an appropriate probability distribution: beta distributions for probabilities and utilities, gamma distributions for costs and utility decrements, and log-normal distributions for RRs. We performed 1,000 iterations. PSA results are presented as cost-effectiveness scatterplots and cost-effectiveness acceptability curves.

### Model validation

Model validation was performed in accordance with recommended good practice for health economic modeling (Author anonymous, 2013). Internal validity was assessed by confirming that transition probabilities from each health state summed to 1. Face validity was evaluated by clinical experts. For external validity, the model-predicted probability of fatal MI (9.03%) was consistent with the in-hospital STEMI mortality rate reported in the Thai ACS Registry (9.05%) (UPDATE Thai ACS Registry for Save Thais, 2024, 2024), and the model-predicted life expectancy of the comparator arm (11.32 years from age 62) was consistent with Thai national life table estimates, supporting the plausibility of the model’s mortality predictions.

## Results

### Base-case analysis

The base-case costs and outcomes for both strategies are summarized in Table 2. From a societal perspective, adding ezetimibe to moderate-intensity statin therapy increased quality-adjusted life-years (QALYs) by 0.2026 and increased total costs by 31,470 THB (891.6 USD), resulting in an incremental cost-effectiveness ratio (ICER) of 155,312 THB/QALY (4,400.4 USD/QALY) gained. From the healthcare provider’s perspective, the addition of ezetimibe increased total costs by 30,178 THB (855 USD), resulting in an ICER of 148,934 THB/QALY (4,219.7 USD/QALY) gained. Using the Thai willingness-to-pay (WTP) threshold of 160,000 THB/QALY (4,533 USD/QALY), ezetimibe added to moderate-intensity statin therapy met the Thai WTP threshold in the base-case analysis compared with moderate-intensity statin therapy alone (Table 3). Applying a half-cycle correction changed the ICERs by −0.14% and −0.15% from the societal and healthcare provider perspectives, respectively (Supplementary Appendi 3, Supplementary Tables S4–S5).

**TABLE 2:** Base-case results.

Cost and outcome parameters	Moderate-intensity statin alone	Ezetimibe + moderate-intensity statin	Incremental
**Drug costs (THB (USD))**	**3,306 (93.7)**	**23,372 (662.1)**	**+20,066 (568.5)**
Cost of moderate-intensity statin	3,306 (93.7)	3,376 (95.6)	+70 (2.0)
Cost of ezetimibe	-	19,996 (566.5)	+19,996 (566.5)
**ACS treatment costs (THB (USD))**	**395,889 (11,216.4)**	**408,656 (11,578.5)**	**+12,767 (361.7)**
ACS first year	60,961 (1727.2)	61,157 (1732.7)	+196 (5.6)
ACS second year and onward	334,928 (9489.3)	345,499 (9788.8)	+10,571 (299.5)
**Non-fatal MI costs (THB (USD))**	**24,668 (699.0)**	**24,452 (692.8)**	**−216 (-6.1)**
Non-fatal MI first year	2,434 (69)	2,129 (60.3)	−305 (−8.6)
Non-fatal MI second year and onward	22,234 (629.9)	22,323 (632.5)	+89 (2.5)
**Non-fatal stroke costs (THB (USD))**	**6,424 (182.0)**	**5,985 (169.6)**	**−439 (-12.4)**
Non-fatal stroke first year	386 (10.9)	332 (9.4)	−54 (−1.5)
Non-fatal stroke second year and onward	6,038 (171.1)	5,653 (160.2)	−385 (−10.9)
**Total direct medical cost (THB (USD))**	**430,287 (12,191.1)**	**460,465 (13,046.1)**	**+30,178 (855.0)**
**Direct non-medical cost (THB (USD))**	**59,488 (1,685.4)**	**60,780 (1,722.0)**	**+1,292 (36.6)**
Direct non-medical cost first year	3,552 (100.6)	3,552 (100.6)	0 (0.0)
Direct non-medical cost second year and onward	55,936 (1,584.8)	57,228 (1,621.4)	+1,292 (36.6)
Total cost			
** Societal perspective**	**489,774 (13,876.5)**	**521,245 (14,768.1)**	**+31,470 (891.6)**
** Healthcare provider perspective**	**430,287 (12,191.1)**	**460,465 (13,046.1)**	**+30,178 (855.0)**
Outcomes			
LYs	11.3216	11.5612	+0.2396
QALYs	8.5772	8.7798	+0.2026

Costs are presented in 2024 Thai baht (THB) with USD, values in parentheses, calculated at an exchange rate of 35.2952 THB/USD.

*D*irect non-medical costs are included only from the societal perspective. The willingness-to-pay threshold was 160,000 THB/QALY (4,533 USD/QALY).

*A*CS, acute coronary syndrome; LYs, life-years; MI, myocardial infarction; QALYs, quality-adjusted life-years; THB, thai baht.

Bold values represent subtotals or summary outcome rows.

**TABLE 3:** Incremental cost-effectiveness ratios results.

Cost-effectiveness outcomes	Societal perspective	Healthcare provider perspective
Incremental cost (THB (USD))	31,470 (891.6)	30,178 (855.0)
Incremental LYs	0.2396	0.2396
Incremental QALYs	0.2026	0.2026
**ICER (THB/LYs (USD/LYs))**	**131,338 (3,721.1)**	**125,945 (3,568.3)**
**ICER (THB/QALYs (USD/QALYs))**	**155,312 (4,400.4)**	**148,934 (4,219.7)**

Costs are presented in 2024 Thai baht (THB) with USD, values in parentheses, calculated at an exchange rate of 35.2952 THB/USD.

ICER, incremental cost-effectiveness ratio; LYs, life-years; QALYs, quality-adjusted life-years; THB, thai baht.

Bold values represent subtotals or summary outcome rows.

### One-way sensitivity analysis

**FIGURE 2:**
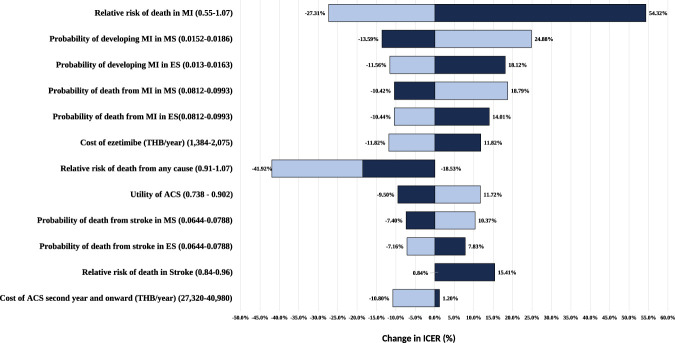
Tornado diagram for one-way sensitivity analysis. ACS, acute coronary syndrome; ES, ezetimibe plus moderate-intensity statin; MI, myocardial infarction; MS, moderate-intensity statin alone; THB, Thai baht.

One-way sensitivity analysis (Figure 2) identified the relative risk of death from myocardial infarction (MI) (range: 0.55–1.07) as the most influential parameter, with ICER changes ranging from −27.31% to +54.32%. The relative risk of all-cause death (range: 0.91–1.07) also materially affected the ICER (−41.92% to −18.53%). Other parameters, including the cost of ezetimibe, utility in the post-ACS “no further event” state, and probabilities of MI and stroke, had moderate to minimal effects on the ICER, and the base-case conclusion remained unchanged across all tested ranges.

### Probabilistic sensitivity analysis

**FIGURE 3:**
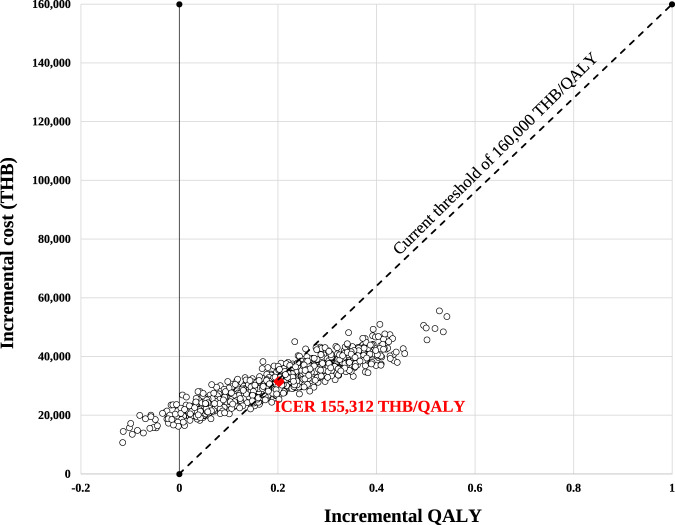
Cost-effectiveness scatterplots. The willingness-to-pay threshold was 160,000 THB/QALY (4,533 USD/QALY). ICER, incremental cost-effectiveness ratio; QALY, quality-adjusted life-year; THB, Thai baht.

**FIGURE 4:**
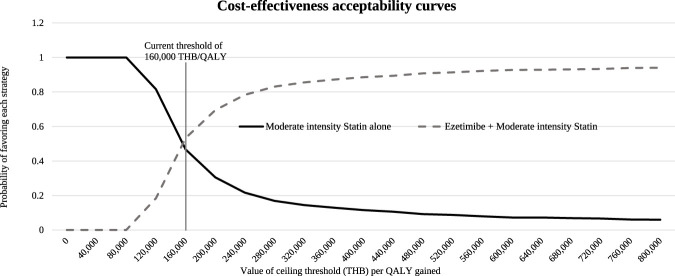
Cost-effectiveness acceptability curves. The willingness-to-pay threshold was 160,000 THB/QALY (4,533 USD/QALY). ICER, incremental cost-effectiveness ratio; QALY, quality-adjusted life-year; THB, Thai baht.

Cost-effectiveness scatterplots based on 1,000 Monte Carlo simulations (Figure 3) showed that 96.3% of iterations fell in the northeast quadrant, indicating that ezetimibe added to moderate-intensity statin therapy was more effective and more costly than statin therapy alone. At the predefined WTP threshold of 160,000 THB/QALY (4,533 USD/QALY), the probability that the intervention was cost-effective was 56.8% (Figure 4).

## Discussion

This economic evaluation assessed the value of adding ezetimibe to moderate-intensity statin therapy compared with moderate-intensity statin therapy alone for secondary prevention among patients with ACS who are intolerant to high-intensity statins in Thailand. Although the base-case ICER (155,312 THB/QALY) was below the national willingness-to-pay (WTP) threshold, the difference was small, and probabilistic sensitivity analysis (PSA) showed only a 56.8% probability of cost-effectiveness at the predefined threshold. Accordingly, the findings support a borderline conclusion regarding cost-effectiveness, and decision-makers should explicitly consider this uncertainty when evaluating reimbursement of ezetimibe for this indication.

Our findings are broadly consistent with evidence from the United Kingdom, where ezetimibe added to statin therapy for secondary prevention after ACS was reported to be more cost-effective than statin therapy alone (Reckless et al., 2010). In contrast, a study from the United States suggested that the combination strategy was not cost-effective over time horizons of <10 years, although it became cost-effective over a lifetime horizon using a $50,000/QALY threshold (Almalki et al., 2018). In Thailand, a previous study by Kongpakwattana et al. found that adding ezetimibe for secondary prevention in patients with cardiovascular disease (CVD) was not cost-effective compared with statin therapy alone (Kongpakwattana et al., 2019). Differences between that study and the present analysis may be attributable to variations in model structure, population characteristics, statin regimen assumptions, and ezetimibe acquisition costs. Importantly, the clinical scenario evaluated here reflects routine lipid-lowering practice in Thailand: a substantial proportion of Thai patients with ACS receive moderate-intensity statins in real-world care (Buddhari et al., 2020) and do not achieve guideline-recommended LDL-C targets (The Heart Association of Thailand, 2020). Therefore, the ICERs observed from both societal and healthcare provider perspectives suggest that ezetimibe add-on therapy may represent a potentially cost-effective and pragmatically relevant strategy within the Thai healthcare system, while acknowledging the uncertainty highlighted by PSA.

The IMPROVE-IT trial was used as the primary clinical efficacy source for several reasons. First, it remains the only large randomized controlled trial (n = 18,144) designed to evaluate the incremental benefit of adding ezetimibe to moderate-intensity statin therapy in an ACS population (Cannon et al., 2015), which directly matches the clinical question addressed in this analysis. Second, the comparator regimen in IMPROVE-IT (simvastatin 40 mg monotherapy) corresponds to a moderate-intensity statin option available on the Thai NLEM, supporting alignment between trial regimens and the Thai formulary. Third, the mean age of trial participants (approximately 64 years) is close to the mean age reported in the Thai ACS Registry (62 years) (Thai ACS Registry, 2023), supporting the applicability of treatment-effect estimates to the Thai setting. Moreover, key model inputs, including mortality risks and costs, were derived from Thailand-specific sources, and drug costs were based on national pricing information. Consistent with Thai HTA guidance, the median price across brands was used (Author anonymous, 2013).

Several limitations should be acknowledged. First, the model did not allow for concurrent MI and stroke, primarily due to limited evidence to inform joint-event probabilities. However, available data suggest that such co-occurrence is uncommon (approximately 1.6%) (Aggarwal et al., 2021; Alqahtani et al., 2017), and exclusion is unlikely to materially affect incremental comparisons. Second, adverse events and treatment discontinuation were not explicitly modeled. Nevertheless, ezetimibe in combination with statin therapy has a safety and tolerability profile comparable to statin monotherapy (Cannon et al., 2015), suggesting that omission of discontinuation and adverse-event costs would be unlikely to bias results strongly in favor of either strategy. Third, crossover transitions between the non-fatal myocardial infarction (MI) and non-fatal stroke states were not modeled. Thai-specific evidence on the rate of such sequential transitions is unavailable. However, the IMPROVE-IT trial reported an annual ischemic stroke incidence of approximately 0.59% among statin-treated patients with acute coronary syndrome (Bohula et al., 2017), which may be considered a plausible upper bound for this transition probability. Because this assumption was applied symmetrically to both treatment arms, its impact on the incremental cost-effectiveness ratio is expected to be minimal.

Generalizability within Thailand also warrants consideration. Drug acquisition costs were sourced from the national DMSIC database (National Drug Prices, 2023), which reflects the nationwide median price across brands and is therefore intended to be broadly representative. Clinical event risks were derived from national registry and administrative data sources (Update Thai ACS Registry for Save Thais, 2024; Health Information System, 2024), which capture patients across multiple hospital levels and geographic regions. However, access to ezetimibe and the quality of post-ACS care may differ by setting. Future analyses incorporating institution- or region-specific inputs could further strengthen the evidence base for national decision-making.

From a policy perspective, these findings provide additional evidence to inform secondary prevention strategies for ACS, particularly for patients who cannot tolerate high-intensity statin therapy. The approach and results may also be informative for countries with healthcare systems and cost structures similar to Thailand’s.

## Conclusion

This health economic analysis suggests that adding ezetimibe to moderate-intensity statin therapy may be cost-effective compared with moderate-intensity statin monotherapy for secondary prevention among patients with ACS who are intolerant to high-intensity statins in Thailand. Although the base-case ICER (155,312 THB/QALY; 4,400.4 USD/QALY) was slightly below the national threshold, the PSA indicated only a 56.8% probability of cost-effectiveness at the threshold. Therefore, the results should be interpreted with appropriate caution.

## Data Availability

The original contributions presented in the study are included in the article/Supplementary Material, further inquiries can be directed to the corresponding author.
