# Supplementary material for: Cost-utility analysis of add-on ezetimibe to moderate-intensity statin versus moderate-intensity statin alone for secondary prevention in patients with acute coronary syndrome intolerant to high-intensity statin therapy in Thailand
Source: Front Pharmacol. 2026 May 20;17:1813614. doi: 10.3389/fphar.2026.1813614 (PMC13230077; doi:10.3389/fphar.2026.1813614)
Supplement: Supplementary file 1 [file DataSheet1.docx]

**Supplementary Appendix**

**Cost-utility analysis of add-on ezetimibe to moderate intensity statin versus moderate-intensity statin alone for secondary prevention in patients with acute coronary syndrome in Thailand.**

**Supplementary contents**

[Supplementary Appendix 1 Details of the probability of dying and annual mortality rate 3](#_Toc228116665)

[Table S1 Details of the probability of dying between ages x and x+n in 2019(1). 3](#_Toc228116666)

[Table S2 Details of annual mortality rate and probability by age(1, 2). 4](#_Toc228116667)

[Supplementary Appendix 2 Details of input cost parameter 6](#_Toc228116668)

[Table S3 Details of cost and dosage regimens for all interventions considered in this study(3) 6](#_Toc228116669)

[Supplementary Appendix 3 Comparison of base-case results with and without half-cycle correction 7](#_Toc228116670)

[Table S4: Base-case results with and without half-cycle correction 7](#_Toc228116671)

[Table S5: ICER results with and without half-cycle correction. 8](#_Toc228116672)

[Reference 8](#_Toc228116673)

# Supplementary Appendix 1 Details of the probability of dying and annual mortality rate

## Table S1 Details of the probability of dying between ages x and x+n in 2019(1).

| Indicator | Age group | Probability of dying |
| --- | --- | --- |
| 0 | &lt;1 year | 0.007695335 |
| 1 | 1-4 years | 0.000322633 |
| 5 | 5-9 years | 0.00032789 |
| 10 | 10-14 years | 0.000511851 |
| 15 | 15-19 years | 0.000884612 |
| 20 | 20-24 years | 0.001196805 |
| 25 | 25-29 years | 0.001610319 |
| 30 | 30-34 years | 0.002091867 |
| 35 | 35-39 years | 0.002547962 |
| 40 | 40-44 years | 0.003060661 |
| 45 | 45-49 years | 0.003771536 |
| 50 | 50-54 years | 0.004965394 |
| 55 | 55-59 years | 0.006578711 |
| 60 | 60-64 years | 0.009678037 |
| 65 | 65-69 years | 0.014788079 |
| 70 | 70-74 years | 0.022686733 |
| 75 | 75-79 years | 0.035582914 |
| 80 | 80-84 years | 0.05691562 |
| 85 | 85+ years | 0.123361855 |

## Table S2 Details of annual mortality rate and probability by age(1, 2).

| Age | Annual mortality rate | Probability of All cause Mortality |
| --- | --- | --- |
| 50 | 0.004965394 | 0.009881641 |
| 51 | 0.004965394 | 0.009881641 |
| 52 | 0.004965394 | 0.009881641 |
| 53 | 0.004965394 | 0.009881641 |
| 54 | 0.004965394 | 0.009881641 |
| 55 | 0.006578711 | 0.013071242 |
| 56 | 0.006578711 | 0.013071242 |
| 57 | 0.006578711 | 0.013071242 |
| 58 | 0.006578711 | 0.013071242 |
| 59 | 0.006578711 | 0.013071242 |
| 60 | 0.009678037 | 0.019169948 |
| 61 | 0.009678037 | 0.019169948 |
| 62 | 0.009678037 | 0.019169948 |
| 63 | 0.009678037 | 0.019169948 |
| 64 | 0.009678037 | 0.019169948 |
| 65 | 0.014788079 | 0.029143064 |
| 66 | 0.014788079 | 0.029143064 |
| 67 | 0.014788079 | 0.029143064 |
| 68 | 0.014788079 | 0.029143064 |
| 69 | 0.014788079 | 0.029143064 |
| 70 | 0.022686733 | 0.044359484 |
| 71 | 0.022686733 | 0.044359484 |
| 72 | 0.022686733 | 0.044359484 |
| 73 | 0.022686733 | 0.044359484 |
| 74 | 0.022686733 | 0.044359484 |
| 75 | 0.035582914 | 0.068692558 |
| 76 | 0.035582914 | 0.068692558 |
| 77 | 0.035582914 | 0.068692558 |
| 78 | 0.035582914 | 0.068692558 |
| 79 | 0.035582914 | 0.068692558 |
| 80 | 0.05691562 | 0.107591454 |
| 81 | 0.05691562 | 0.107591454 |
| 82 | 0.05691562 | 0.107591454 |
| 83 | 0.05691562 | 0.107591454 |
| 84 | 0.05691562 | 0.107591454 |
| 85 | 0.123361855 | 0.218643455 |
| 86 | 0.123361855 | 0.218643455 |
| 87 | 0.123361855 | 0.218643455 |
| 88 | 0.123361855 | 0.218643455 |
| 89 | 0.123361855 | 0.218643455 |
| 90 | 0.123361855 | 0.218643455 |
| 91 | 0.123361855 | 0.218643455 |
| 92 | 0.123361855 | 0.218643455 |
| 93 | 0.123361855 | 0.218643455 |
| 94 | 0.123361855 | 0.218643455 |
| 95 | 0.123361855 | 0.218643455 |
| 96 | 0.123361855 | 0.218643455 |
| 97 | 0.123361855 | 0.218643455 |
| 98 | 0.123361855 | 0.218643455 |
| 99 | 0.123361855 | 0.218643455 |
| 100 | 1 | 0.864664717 |

# Supplementary Appendix 2 Details of input cost parameter

## Table S3 Details of cost and dosage regimens for all interventions considered in this study(3)

| Treatment | Dosage | Trade name | Median cost per unit (THB) | Annual median cost (THB) | Cost per year (THB) |
| --- | --- | --- | --- | --- | --- |
| Simvastatin 40 mg | OD | BESTATIN | 0.8 | 0.80 | 292 |
|  | OD | EUCOR | 0.7918 |  |  |
|  | OD | ZIMVA | 0.963 |  |  |
|  | OD | BESTATIN | 0.8 |  |  |
|  | OD | EUCOR | 0.7918 |  |  |
|  | OD | ZIMMEX | 0.9 |  |  |
| Ezetimibe 10 mg | OD | EZETIMIBE GPO | 2.996 | 4.74 | 1,729.58 |
|  | OD | ZETIA | 15.2296667 |  |  |
|  | OD | EZETIMIBE SANDOZ | 5.35 |  |  |
|  | OD | EZOMIB | 4.298 |  |  |
|  | OD | MIBEAZ | 2.996 |  |  |
|  | OD | EZENTIA | 4.73857143 |  |  |
|  | OD | EZENTIA | 4.73857143 |  |  |
|  | OD | EZB | 3.26666667 |  |  |
|  | OD | EZETROL | 15.2296667 |  |  |
|  | OD | EZETIMIBE SANDOZ | 5.35 |  |  |
|  | OD | EZOMIB | 4.298 |  |  |

THB, Thai baht; OD, Once daily

# Supplementary Appendix 3 Comparison of base-case results with and without half-cycle correction

## Table S4: Base-case results with and without half-cycle correction

| ***Parameter*** | ***Base-case results*** | | ***Results with half-cycle correction*** | |
| --- | --- | --- | --- | --- |
|  | **Moderate-intensity statin alone** | **Ezetimibe + Moderate-intensity statin** | **Moderate-intensity statin alone** | **Ezetimibe + Moderate-intensity statin** |
| **Drug costs** |  |  |  |  |
| Cost of moderate-intensity statin | 3,305 | 3,374 | 3,306 | 3,376 |
| Cost of ezetimibe | - | 19,981 | - | 19,996 |
| **Treatments cost (THB)** |  |  |  |  |
| ACS first year | 60,961 | 61,156 | 60,961 | 61,157 |
| ACS second year and onward | 334,926 | 345,496 | 334,928 | 345,499 |
| Non-fatal MI first year | 2,434 | 2,128 | 2,434 | 2,129 |
| Non-fatal MI second year and onward | 22,184 | 22,258 | 22,234 | 22,323 |
| Non-fatal stroke first year | 386 | 331 | 386 | 332 |
| Non-fatal stroke second year and onward | 6,019 | 5,633 | 6,038 | 5,653 |
| **Direct non-medical cost (THB)** |  |  |  |  |
| Direct non-medical cost first years | 3,551 | 3,552 | 3,552 | 3,552 |
| Direct non-medical cost second year and onward | 55,910 | 57,195 | 55,936 | 57,228 |
| **Total Cost** | **489,677** | **521,113** | **489,774** | **521,245** |
| **Outcomes** |  |  |  |  |
| Life years | 11.3169 | 11.5553 | 11.3216 | 11.5612 |
| QALY | 8.5749 | 8.7770 | 8.5772 | 8.7798 |

## Table S5: ICER results with and without half-cycle correction.

| **ICER** | ***Base-case results*** | ***Results with half-cycle correction*** | ***% Change*** |
| --- | --- | --- | --- |
| **Societal perspective** |  |  |  |
| THB/LYs | **131,838** | **131,338** | -0.37925 |
| THB/QALYs | **155,527** | **155,312** | -0.13824 |
| **Healthcare provider perspective** |  |  |  |
| THB/LYs | **126,445** | **125,945** | -0.39543 |
| THB/QALYs | **149,164** | **148,934** | -0.15419 |

# Reference

1. Life tables: Life tables by country Thailand [Internet]. 2020 [cited 25 Jan 2024]. Available from: <https://apps.who.int/gho/data/view.searo.61640?lang=en>.

2. Nikolic E, Janzon M, Hauch O, Wallentin L, Henriksson M, Group PHES. Cost-effectiveness of treating acute coronary syndrome patients with ticagrelor for 12 months: results from the PLATO study. Eur Heart J. 2013;34(3):220-8.

3. National Drug Prices [Internet]. Drug And Medical Supply Information Center (DMSIC), Ministry of Public Health. 2023 [cited 25 Jan 2023]. Available from: <http://dmsic.moph.go.th/>.
